# Differential effects on BAFF and APRIL levels in rituximab-treated patients with systemic lupus erythematosus and rheumatoid arthritis

**DOI:** 10.1186/ar2076

**Published:** 2006-11-08

**Authors:** Therese Vallerskog, Mikael Heimbürger, Iva Gunnarsson, Wei Zhou, Marie Wahren-Herlenius, Christina Trollmo, Vivianne Malmström

**Affiliations:** 1 Rheumatology Unit, Department of Medicine Solna, Karolinska Institutet, CMM L8:04, Karolinska Hospital, SE-171 76 Stockholm, Sweden

## Abstract

The objective of this study was to investigate the interaction between levels of BAFF (B-cell activation factor of the tumour necrosis factor [TNF] family) and APRIL (a proliferation-inducing ligand) and B-cell frequencies in patients with systemic lupus erythematosus (SLE) and rheumatoid arthritis (RA) treated with the B-cell-depleting agent rituximab. Ten patients with SLE were treated with rituximab in combination with cyclophosphamide and corticosteroids. They were followed longitudinally up to 6 months after B-cell repopulation. Nine patients with RA, resistant or intolerant to anti-TNF therapy, treated with rituximab plus methotrexate were investigated up to 6 months after treatment. The B-cell frequency was determined by flow cytometry, and serum levels of BAFF and APRIL were measured by enzyme-linked immunosorbent assays. BAFF levels rose significantly during B-cell depletion in both patient groups, and in patients with SLE the BAFF levels declined close to pre-treatment levels upon B-cell repopulation. Patients with SLE had normal levels of APRIL at baseline, and during depletion there was a significant decrease. In contrast, patients with RA had APRIL levels 10-fold higher than normal, which did not change during depletion. At baseline, correlations between levels of B cells and APRIL, and DAS28 (disease activity score using 28 joint counts) and BAFF were observed in patients with RA. In summary, increased BAFF levels were observed during absence of circulating B cells in our SLE and RA patient cohorts. In spite of the limited number of patients, our data suggest that BAFF and APRIL are differentially regulated in different autoimmune diseases and, in addition, differently affected by rituximab treatment.

## Introduction

Systemic lupus erythematosus (SLE) and rheumatoid arthritis (RA) are chronic inflammatory rheumatic diseases, in which autoantibodies are part of the early disease manifestations. A pathogenic involvement of B cells is well documented in SLE and implicated in RA. Rituximab is a chimeric monoclonal antibody that depletes B cells by targeting CD20, a surface molecule expressed exclusively on B cells. After rituximab infusion, circulating B cells are rapidly depleted and remain absent for months. Although originally developed to treat B-cell lymphomas, it has also been used successfully in various autoimmune diseases, including SLE and RA (reviewed by Eisenberg [1]).

Recently, two closely related cytokines that belong to the tumour necrosis factor (TNF) family and that are important for B-cell development and function were described: BAFF (B-cell activation factor of the TNF family, BlyS, THANK, TALL-1, TNFSF13b, zTNF-4) and APRIL (a proliferation-inducing ligand, TNFSF13a) [2]. They share two receptors, BCMA (B-cell maturation antigen) and TACI (transmembrane activator and CAML [calcium-modulating cyclophilin ligand] interactor), which are found mainly on B cells and plasma cells (reviewed by Ng and colleagues [3] and Schneider [4]). The third receptor specific for BAFF, BAFF-R (BAFF receptor, BR3), is found mainly on B cells, plasma cells, but also on some subsets of T cells [3,4]. So far, APRIL has no specific receptor of its own, but it has been shown to bind proteoglycans [5]. With the above receptors expressed mainly on B cells, these cells are the major consumers of these cytokines.

BAFF is produced constitutively by stromal cells within lymphoid organs [6] and is inducible by cells of myeloid origin (monocytes, macrophages, neutrophils, and dendritic cells) and also by osteoclasts (reviewed by Ng and colleagues [3] and Dillon and colleagues [7]). APRIL is produced mainly by the same cells as BAFF [3,7]. It was recently demonstrated that some B cells also produce BAFF; examples are tonsillar germinal centre B cells, Epstein-Barr virus-infected B cells, *in vitro *anti-immunoglobulin (Ig)- and CD40L-activated B cells, and non-Hodgkin lymphoma B cells. [8-11].

Overexpression of BAFF in mice leads to autoimmunity with SLE-like symptoms, while mature B cells are lacking in BAFF-deficient mice [2]. In contrast, mice deficient for APRIL have normal peripheral B-cell populations but increased numbers of effector/memory T cells. Mice overexpressing APRIL have an increased frequency of B cells and an increased level of serum IgM [7].

Abnormal levels of both BAFF and APRIL have been observed in patients with SLE, RA, and Sjögren's syndrome [12-16]. With regard to the strong impact of BAFF and APRIL on B-cell development/function and the deviated levels in SLE and RA, it was of interest to study the effects of rituximab-induced B-cell depletion on these cytokines. We chose to follow changes in BAFF and APRIL serum levels after rituximab therapy in 10 patients with SLE and nine patients with RA. In all patients, BAFF levels increased significantly during B-cell depletion. In contrast, APRIL levels in SLE started out normal and decreased, whereas in RA the levels were high and remained unaffected by rituximab. These data, based on a limited number of patients, suggest that BAFF and APRIL are differentially regulated in different autoimmune diseases and, in addition, differently affected by rituximab-induced B-cell depletion.

## Materials and methods

### Patients and controls

Ten patients with refractory and active SLE (as defined by the American College of Rheumatology criteria) [17] received four weekly infusions (375 mg/m^2^) of rituximab (Mabthera, Rituxan; Roche, Basel, Switzerland). Cyclophosphamide (0.5 g/m^2^) was included at the first and fourth occasions, and corticosteroids were given through the whole treatment. After the fourth infusion, no therapy other than corticosteroids was given until repopulation occurred. All patients showed a clinical response measured as SLAM (systemic lupus activity measure) score (Table 1 and [18]) or in histopathological analysis of kidney biopsies (I. Gunnarsson, personal communication).

Nine patients with active RA, non-responders (did not reach ACR20 [American College of Rheumatology 20% response criteria]) or intolerant to anti-TNF-α therapy (adverse reactions), received two rituximab infusions (1,000 mg/infusion) with an interval of 14 days in combination with oral methotrexate (10 to 20 mg/week). Corticosteroids were given throughout the treatment. The clinical response at B-cell depletion and 6 months is presented in Table 2.

All patients were recruited from the Rheumatology Clinic at the Karolinska University Hospital, Stockholm, Sweden. Thirteen non-treated healthy subjects, median age 60 years (range 20 to 85 years), were used as controls. This study was performed after human ethics approval, and informed consent was obtained from all contributing individuals.

### B-cell-related time points for analysis

With the known variability in time to B-cell return after rituximab treatment and with the aim of correlating changes of BAFF and APRIL levels to the absence/presence of circulating B cells, we chose to study serum levels of BAFF and APRIL in the patients with SLE at B-cell-related time points: that is, (a) at baseline (before treatment), (b) at depletion (B cells less than 0.5% of lymphocytes and less than 0.01 × 10^9 ^per litre of blood), (c) at repopulation (when B cells constitute a significant number of lymphocytes [greater than 1.0%]), and (d) at recovery (the next following sample [2 to 6 months] after repopulation). Three patients were followed for 12 months after repopulation and two patients for 24 months after repopulation. Patients with RA were analysed (a) at baseline (before treatment), (b) at depletion (B cells less than 0.5% of lymphocytes and less than 0.01 × 10^9 ^per litre of blood), and (c) 6 months after baseline.

### Serum levels of BAFF and APRIL

Serum levels of BAFF were measured by an enzyme-linked immunosorbent assay (ELISA) kit from R&D Systems, Inc. (Minneapolis, MN, USA), and serum levels of APRIL were measured by an ELISA kit from Bender MedSystems GmbH (Vienna, Austria). No confounding effect of rheumatoid factor on APRIL levels was observed. Samples were analysed in duplicates, and the mean coefficient of variation was 8.3% in the BAFF assay and 15.4% in the APRIL assay. Both kits were used according to the manufacturers' instructions.

### Levels of B cells

The clinical immunology and clinical pathology laboratories at Karolinska University Hospital analysed the B-cell frequencies and numbers (CD19^+^) according to clinical routine by flow cytometry.

### Statistical analysis

Statistical analysis was performed using GraphPad Prism 3.03 (GraphPad Software, Inc., San Diego, CA, USA) and Statistica 7.1 (StatSoft, Inc., Tulsa, OK, USA). For comparison of paired samples before and after treatment, Wilcoxon matched pairs test was used, Mann-Whitney analysis was performed for differences between groups, and Spearman's rank order test was used for correlations of parameters.

## Results

### BAFF levels increased after B-cell depletion in patients with both SLE and RA

**Figure 1:**
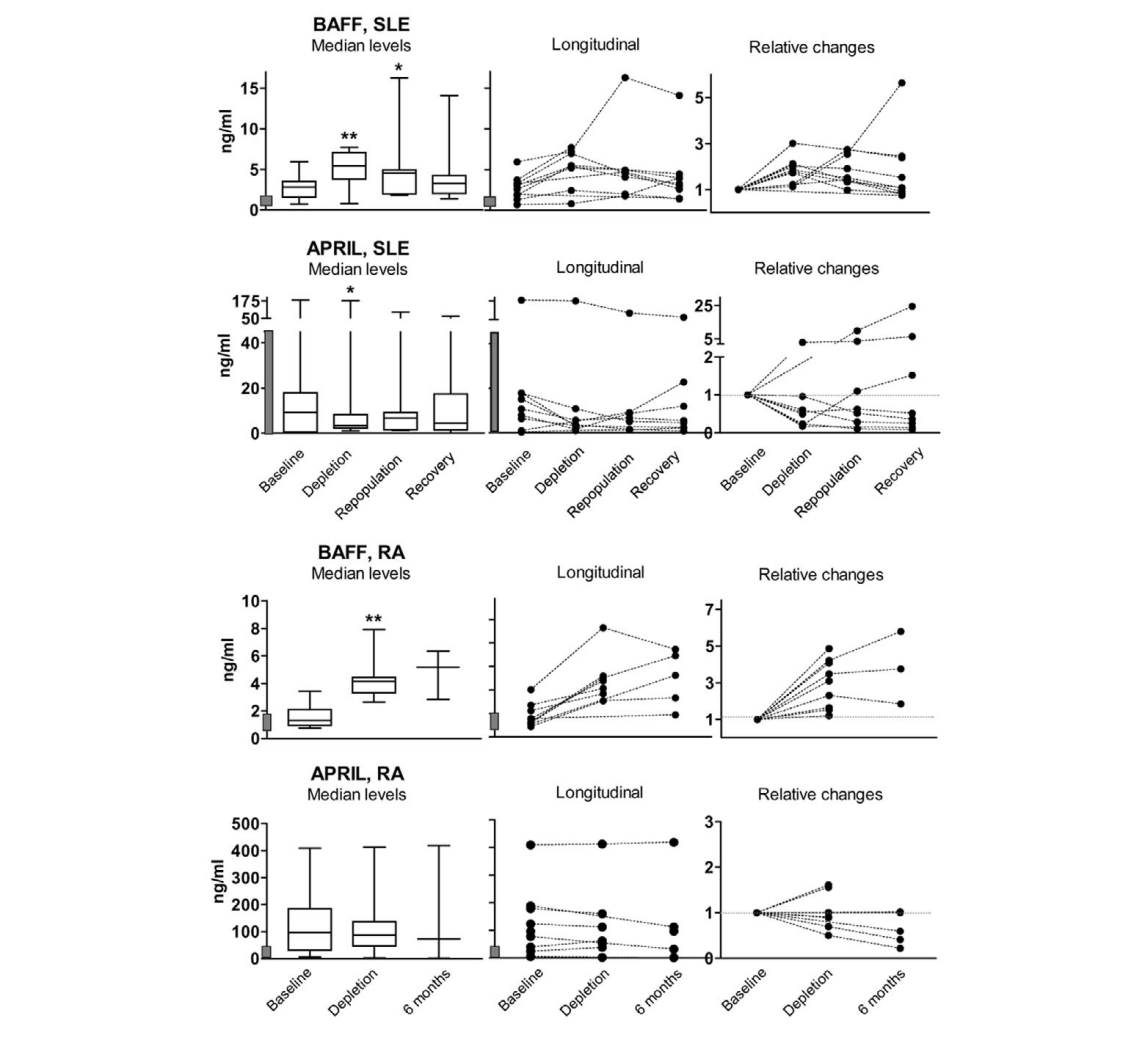
Serum cytokine levels at B-cell-related time points. Left panels: levels of **(a) **BAFF and **(b) **APRIL in patients with systemic lupus erythematosus (SLE) at baseline (*n *= 10), depletion (*n *= 8), repopulation (*n *= 7), and recovery (*n *= 9). A significant increase compared with baseline was observed in BAFF at depletion (*p *< 0.01) and at repopulation (*p *< 0.05). In APRIL, a significant decrease (*p *< 0.05) occurred at depletion compared with baseline. Left panels: levels of **(c) **BAFF and **(d) **APRIL in patients with rheumatoid arthritis (RA) at baseline (*n *= 9), depletion (*n *= 8), and 6 months after infusion (*n *= 5). There was a significant increase (*p *< 0.01) in BAFF levels at depletion compared with baseline. Middle panels: longitudinal levels of BAFF and APRIL in patients with **(a, b) **SLE and **(c, d) **RA; each line corresponds to a different patient. The y-axis has the same scale as the axis in the box-plots. Right panels: relative changes compared with baseline of BAFF and APRIL in patients with **(a, b) **SLE and **(c, d) **RA. Relative change = sample X/baseline sample. The grey bar on the y-axis illustrates the level in healthy controls (*p < 0.05, **p < 0.01). APRIL, a proliferation-inducing ligand; BAFF, B-cell activation factor of the tumour necrosis factor family.

Serum levels of BAFF were followed in 10 patients with SLE before and after rituximab treatment. Upon B-cell depletion, BAFF levels increased significantly relative to baseline (that is, prior to treatment): baseline 2.82 ng/ml (range 0.71 to 5.94 ng/ml) and depletion 5.45 ng/ml (range 0.80 to 7.72 ng/ml, *p *< 0.01) (Figure 1a). In one patient, the BAFF level did not increase until time of repopulation (Figure 1a, Table 1).

When B cells repopulated the circulation (that is, consisted of more than 1% of total lymphocytes), BAFF levels decreased (median 4.54 ng/ml, range 1.79 to 16.3 ng/ml), and at time of recovery, 2 to 6 months after repopulation, the levels decreased further toward pre-treatment values (median 3.30 ng/ml, range 1.41 to 14.1 ng/ml). Twelve months after repopulation, BAFF levels had a median of 2.85 ng/ml (range 1.36 to 3.51 ng/ml, *n *= 3) and remained similar 24 months post-repopulation (2.53 and 2.83 ng/ml, *n *= 2) (Table 1).

**Figure 2:**
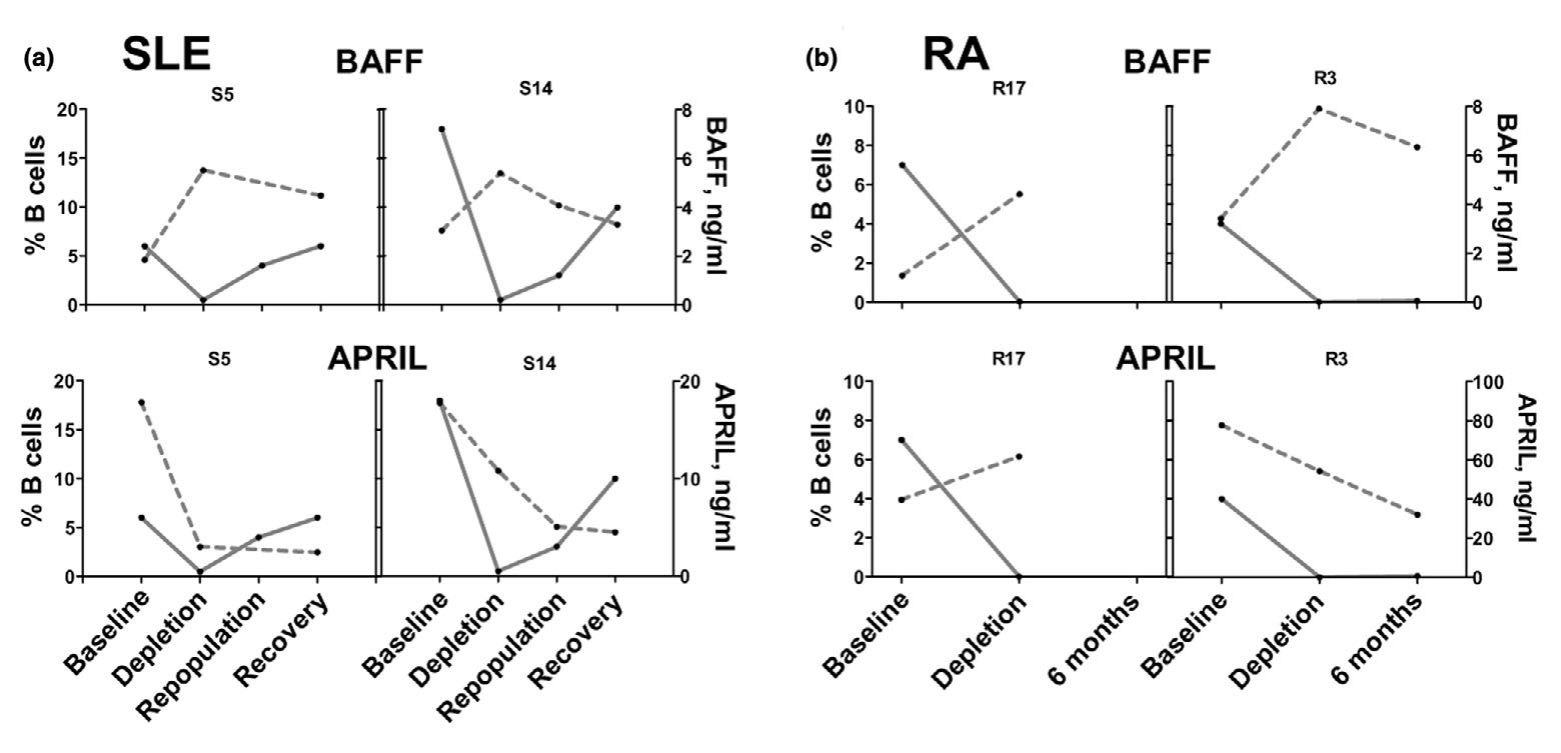
Relation between cytokine levels and B-cell frequency. **(a) **Two patients with systemic lupus erythematosus (SLE) (S5 and S14) representing the relation of B-cell frequency (left y-axis in diagrams) and cytokine levels (right y-axis) of BAFF (top row) and APRIL (bottom row) at B-cell-related time points. Dashed line depicts the cytokine level, and the unbroken line depicts the B-cell frequency. **(b) **Two patients with rheumatoid arthritis (RA) (R17 and R3) representing the relation of B-cell frequency (left y-axis) and levels of cytokines (right y-axis) BAFF (top row) and APRIL (bottom row) at baseline, depletion, and 6 months after treatment. APRIL, a proliferation-inducing ligand; BAFF, B-cell activation factor of the tumour necrosis factor family.

These results were comparable with the nine rituximab-treated patients with RA although a different B-cell depletion protocol was used. Here, at depletion, a threefold increase compared with baseline in BAFF was observed: baseline 1.31 ng/ml (range 0.75 to 3.42 ng/ml) and depletion 4.17 ng/ml (range 2.62 to 7.9 ng/ml, *p *< 0.01) (Figure 1c, Table 2). Only in one patient did the levels of BAFF remain unchanged (R11). In the five patients who were followed for 6 months, the levels remained elevated (median 4.46 ng/ml, range 1.59 to 6.33 ng/ml) (Table 2). At this time point, the B cells had not yet repopulated. Figure 2a and 2b (top row) illustrate levels of BAFF and B-cell frequency in two SLE and two RA patients at the B-cell-related time points.

The healthy subjects had a median of 0.81 ng/ml of BAFF (range 0.56 to 1.67 ng/ml, indicated as grey bars on y-axis in the diagrams in Figure 1). Before treatment, there was a significant difference (*p *< 0.001) in BAFF levels between patients with SLE and healthy controls, whereas there was no significant difference between the patients with RA and healthy controls.

### APRIL levels decreased during B-cell depletion in patients with SLE

In contrast to the observed increase in BAFF levels, APRIL decreased significantly from a median of 9.25 ng/ml (range 0 to 182.3 ng/ml) to 3.34 ng/ml (range 0.99 to 175.7 ng/ml) during B-cell depletion in our SLE cohort (*p *< 0.05) (Figure 1b, Table 1). This occurred in all but one patient (S19). At repopulation and recovery, the levels were still below baseline levels (median 6.65 and 4.49 ng/ml, respectively). Levels of APRIL remained low 12 and 24 months after repopulation (median 6.46 ng/ml, range 0.86 to 40.9 ng/ml [*n *= 3] and 1.24 and 23.8 ng/ml [*n *= 2], respectively) (Table 1). In healthy subjects, the range was 0 to 46.6 ng/ml (median 4.68 ng/ml) and is indicated as grey bars on y-axis in the diagrams in Figure 1.

In contrast to the normal levels of APRIL in the patients with SLE, the patients with RA had significantly higher levels of APRIL at baseline (*p *< 0.05) (median 96.7 ng/ml, range 5.04 to 409 ng/ml) (Figure 1d, Table 2), and also had higher levels than the healthy controls (*p *< 0.001). Upon B-cell depletion, no significant changes were observed (median 86.7 ng/ml, range 2.53 to 413 ng/ml), and in the five patients followed for 6 months post-treatment, the median was 97.1 ng/ml (range 1.12 to 419 ng/ml) (Table 2). On an individual level, five of nine patients downregulated APRIL levels during depletion, two upregulated, and two had unchanged levels of APRIL at depletion. Analysis of APRIL levels in 13 healthy subjects demonstrated that the difference between patients with SLE and RA was not due to differences in age distribution (data not shown).

**Figure 3:**
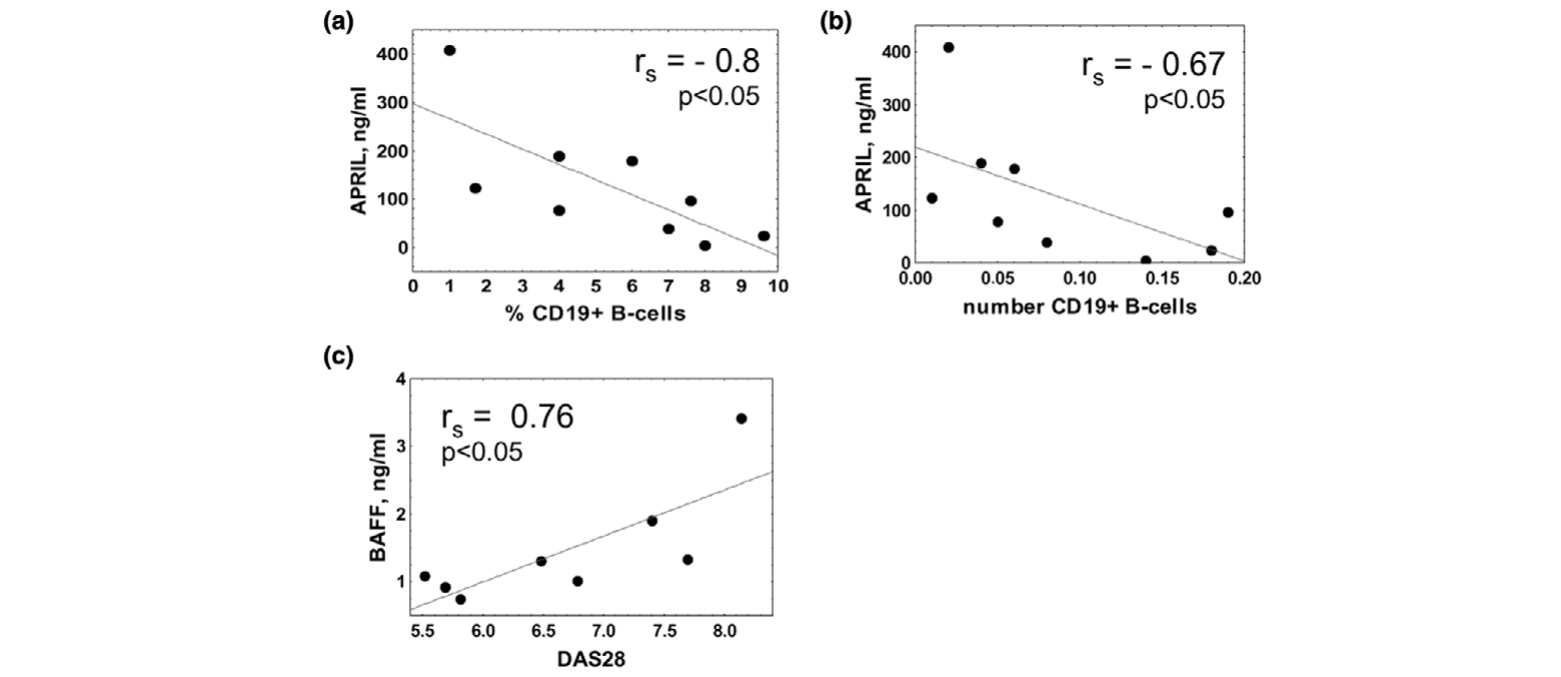
Correlations of BAFF (B-cell activation factor of the tumour necrosis factor family) and APRIL (a proliferation-inducing ligand). **(a) **Negative correlation of the B-cell frequency and APRIL levels in serum at baseline in patients with rheumatoid arthritis (RA) (Spearman r [r_s_] = -0.8, *p *< 0.05). **(b) **There was also a correlation between the number of B cells and levels of APRIL in serum at baseline in the patients with RA (r_s _= -0.67, *p *< 0.05). **(c) **Moreover, a correlation between the disease activity score using 28 joint counts (DAS28) and circulating levels of BAFF at baseline in patients with RA was found (r_s _= 0.76, *p *< 0.05). Each dot represents a different patient, and the line illustrates the slope of r.

After B-cell depletion, the levels of APRIL paralleled the frequency of B cells in most patients with SLE (Figure 2a, bottom row). In contrast, different patterns were observed in patients with RA as illustrated in Figure 2b (bottom row). There was a significant negative correlation between the B-cell frequency and APRIL serum levels (Spearman r [r_s_] = -0.80, *p *< 0.05) in the patients with RA before treatment (Figure 3a). This was also found for the number of B cells and serum levels of APRIL (r_s _= -0.67, *p *< 0.05) in the same patients (Figure 3b). We also observed a positive correlation between disease activity score using 28 joint counts (DAS28) in patients with RA and circulating levels of BAFF (r_s _= 0.76, *p *< 0.05) (Figure 3c). This correlation was valid for the baseline samples only, but at no other time points. No correlations between measured parameters were found in the patients with SLE.

## Discussion

This is the first study to demonstrate both an increase in BAFF levels in patients with SLE and a differential effect on APRIL in patients with SLE and RA treated with the B-cell-depleting agent rituximab. BAFF levels increased significantly after B-cell depletion and decreased upon repopulation in our SLE cohort. Similar changes have been demonstrated in patients with RA and primary Sjögren's syndrome treated with different rituximab protocols, suggesting that this pattern is a consequence of the B-cell depletion *per se *(Figure 1a,c; data by Cambridge and colleagues [19] and Seror and colleagues [20]).

### BAFF and APRIL before treatment

In RA, the levels of BAFF were close to normal before treatment (at baseline) in the majority of patients (*n *= 9), and three patients (33%) had higher-than-normal levels. This is in accordance with previously published studies by Cheema and colleagues [21], who describe increased levels of BAFF in 22% of patients (15 of 67) with RA, and Groom and colleagues [22], who report increased BAFF levels in 19% of their RA patient cohort. In most patients with SLE, the levels of BAFF were above normal before treatment, which also agrees with data from earlier studies [12-16]. These increased BAFF levels in SLE patients could be one contributing factor in the observed increased frequency of plasmablasts, as these cells express both BCMA and BAFF-R [23-25]. Also, the increased levels of BAFF could contribute to survival of autoreactive B cells that would otherwise succumb to negative selection. This has also been suggested by Pers and colleagues [15] and Kalled [26]. Levels of APRIL were within the normal range in our SLE cohort. In the literature, there are contrasting reports regarding APRIL levels in SLE. Stohl and colleagues [27] describe normal levels in a majority of patients (*n *= 68), whereas Koyama and colleagues [28] report increased serum levels in their patients (*n *= 48). In our small patient cohort, we did not find any correlations between measured parameters (SLAM, frequency of B cells, number of B cells, BAFF, and APRIL) (Table 1) at any time point.

In contrast to the SLE patients, the patients with RA had on average 10-fold higher levels of APRIL in serum. However, in three of nine patients, we measured normal levels. There are a few publications regarding APRIL in patients with RA. Koyama and colleagues [28] report normal serum levels in a cohort of 21, three of whom were above normal. Tan and colleagues [29] show higher APRIL levels in synovial fluid compared with serum. In addition, Seyler and colleagues [13] studied mRNA levels of APRIL in synovial biopsies, in which the samples were classified as germinal centre synovitis, aggregate synovitis, or diffuse synovitis, ranking inflammatory activity from severe to mild, respectively. The tissue expression of APRIL mRNA was the highest in germinal centre-positive synovitis and the lowest in diffuse synovitis. The authors suggest that APRIL mRNA levels correlate with the variability of tissue B-cell function.

At this point, we can only speculate why we see different levels of BAFF and APRIL in our two patient cohorts. The high circulating levels of APRIL in RA are striking even though our patient cohort is small. From the data by Seyler and colleagues [13] as described above, we could speculate that all RA patients included in our study have germinal centre synovitis. Another hypothesis is that different cytokine profiles induce different amounts of BAFF and APRIL. Patients with SLE have increased levels of interferon (IFN)-α and IL-10 [30,31], and these cytokines induce production of BAFF while APRIL expression is upregulated by IFN-γ and IFN-α (reviewed by Ng and colleagues [3]). BAFF and APRIL are also probably produced by different cell subsets in RA and SLE. Osteoclasts derived from the inflamed RA joint have been shown to be good producers of APRIL [32]. It has been shown that synovial fluid from patients with active RA contains high levels of BAFF and APRIL, probably locally produced in the joint by neutrophils, dendritic cells, and macrophages [13,29]. Additionally, fibroblast-like synoviocytes secrete BAFF after stimulation with IFN-γ and TNF-α, which are known to be effector cytokines in the RA joint [33]. Thus, different cytokine milieus and different cells producing BAFF and APRIL in the two diseases could contribute to this divergent finding. Previous treatment regimens could also provide clues to the different levels of BAFF and APRIL in patients with SLE and RA. In this context, already at baseline, our cohort of patients with SLE were treated with cyclophosphamide and our patients with RA with methotrexate and/or anti-TNF-α therapy.

Interestingly, despite our small RA patient group, before treatment we found several statistical correlations, which however need to be confirmed in larger patient cohorts. Also, the biological significance of these correlations, a positive correlation between DAS28 and serum levels of BAFF and negative correlations between levels of APRIL and B-cell frequency and number, are now subject to further investigations.

### Effects on BAFF and APRIL after B-cell depletion

During B-cell depletion upon rituximab treatment, levels of BAFF increased in both patients with SLE and RA. That this increase occurred despite different treatment protocols suggests that this change is likely to be a consequence of the B-cell depletion *per se*. Similar results have been presented in two other studies of rituximab-induced B-cell depletion in rheumatic patients, one by Cambridge and colleagues [19] in patients with RA and the other by Seror and colleagues [20] in patients with Sjögren's syndrome. These results support a constitutive expression of BAFF by stromal cells in lymphoid organs [6,34]. The results also indicate that there is no immediate negative regulation of BAFF secretion when the main BAFF consumers (the B cells) are absent or significantly reduced in numbers, as has been demonstrated in the murine setting [6]. A recent report, however, suggests a delayed regulation of BAFF mRNA transcription in rheumatic patients after rituximab treatment [35].

Regarding APRIL, this is the first study to measure potential changes in concentration upon rituximab-induced B-cell depletion. Different patterns were observed in the two patient cohorts: a significant decrease occurred after B-cell depletion in the patients with SLE, whereas in the patients with RA we observed a scattered pattern. Thus, differential effects were seen in changes of BAFF and APRIL upon treatment. This has also been reported after high-dose corticosteroid treatment of patients with SLE: levels of APRIL remained the same before and after treatment, whereas BAFF levels decreased [27]. Our data warrant further extended and mechanistic studies to elucidate the regulation of BAFF and APRIL, including their receptor expression due to effects of different treatments in the different rheumatic diseases.

One concern with the increased availability of BAFF, even if only temporary, is the risk of an increased output of autoreactive B cells. Such an effect has been demonstrated in BAFF transgenic mice, especially under lymphopenic conditions [2,34,36]. Autoreactive B cells are normally eliminated by B-cell-receptor-induced apoptosis during negative selection in the periphery. However, this could be inhibited by the increased availability of BAFF, which by binding to the BAFF-R induces upregulation of anti-apoptotic proteins [4,34]. Another concern is the association of increased BAFF and APRIL levels with different forms of (non-Hodgkin) lymphomas [9,11,37]. In parallel, data exist on increased risk of lymphomas in patients with SLE and RA [38]. Also, plasmablast survival is likely to be enhanced with high levels of BAFF [24]. Moreover, BAFF induces Mcl-1 expression in plasma cells, which is necessary for their survival in the bone marrow [3,25]. Thus, also plasmablasts and plasma cells could be affected by the increased levels of BAFF after rituximab treatment, which could contribute to re-manifest the disease although that occurs long after B-cell repopulation in most patients. Whether and how APRIL affects plasma cells and their migration to the bone marrow remain to be elucidated. APRIL is believed to be involved by binding syndecan-1 (CD138) and in triggering TACI- and/or BCMA-mediated survival signals [5].

Not only B cells are affected by BAFF and APRIL. There are studies showing effects on T cells also [39] (reviewed by Schneider [4]). T-cell survival can be enhanced when the BAFF-R is upregulated upon activation, Bcl-2 is induced, and apoptosis is prevented. BAFF can also co-stimulate T cells. Moreover, human T cells stimulated with BAFF secrete IFN-γ and IL-2 and upregulate CD25 [4]. Indeed, we found an increase of CD25 on both CD4^+ ^and CD8^+ ^T cells in our cohort of rituximab-treated SLE patients [18]. Our data support the suggestion by Cambridge and colleagues [19] that rituximab-treated patients may benefit from complementary anti-BAFF therapy to temporarily remove excess BAFF.

## Conclusion

In this study, we have demonstrated that BAFF levels increased significantly after B-cell depletion and decreased upon B-cell repopulation in our SLE cohort (*n *= 10) treated with rituximab. The similar changes observed in RA patients (*n *= 9) treated with a different rituximab protocol suggest that this pattern is likely a consequence of the B-cell depletion *per se*. Patients with SLE had normal levels of APRIL at baseline, and during depletion there was a significant decrease. The contrasting results from our RA patient cohort, whose APRIL levels were 10-fold higher than normal and did not change during depletion, suggest that APRIL can be differently regulated in RA patients. Our patient groups are small, so these findings need to be confirmed, but given that the cohorts had defined inclusion criteria they represent homogenous subgroups within their respective diseases. In summary, our data suggest that BAFF and APRIL are differentially regulated in SLE and RA and, in addition, heterogeneously affected by rituximab treatment.

## Abbreviations

APRIL = a proliferation-inducing ligand; BAFF = B-cell activation factor of the tumour necrosis factor family; BAFF-R = B-cell activation factor of the tumour necrosis factor family receptor; BCMA = B-cell maturation antigen; DAS28 = disease activity score using 28 joint counts; ELISA = enzyme-linked immunosorbent assay; IFN = interferon; Ig = immunoglobulin; IL = interleukin; RA = rheumatoid arthritis; r_s _= Spearman r; SLAM = systemic lupus activity measure; SLE = systemic lupus erythematosus; TACI = transmembrane activator and CAML (calcium-modulating cyclophilin ligand) interactor; TNF = tumour necrosis factor.

## Competing interests

The authors declare that they have no competing interests.

## Authors' contributions

TV participated in the study design, acquired, analysed and interpreted data, prepared the manuscript, and performed statistical analysis. MH and IG selected and collected samples and interpreted and provided clinical data. WZ acquired data. MW-H analysed and interpreted data and helped prepare the manuscript. CT and VM participated in the study design, analysed and interpreted data, and prepared the manuscript. CT and VM contributed equally to this study. All authors read and approved the final manuscript.

## Figures and Tables

**Table 1:** Disease activity, B-cell status, BAFF, and APRIL at all time points in patients with SLE

	ID	S4	S5	S7	S11	S13	S14	S16	S17	S19	S20
Age (in years)/gender		27/F	56/F	33/F	50/F	33/F	33/F	19/F	35/F	56/F	51/F
SLAM	Baseline	25	14	7	12	4	22	9	14	12	8
	Depletion	11	13	6	10	n.d.	16	11	12	9	n.d.
	6 months post-treatment	8	11	4	6	4	13	n.d.	9	n.d.	1
Percentage of CD19^+ ^B cells	Baseline	5	6	2	2	15	18	6	8	8	1
	Depletion	bdl	bdl	bdl	bdl	n.d.	bdl	bdl	bdl	bdl	bdl
	Repopulation	5	4		8	11	3	4	8	4	2
	Recovery	2	6		9	18	10	n.d.	8	12	5
	12 months after repopulation	8								11	11
	24 months after repopulation	13				18					
CD19^+ ^B cells × 10^9 ^per litre	Baseline	0.04	0.04	0.01	0.01	0.23	0.05	0.03	0.05	0.05	0.01
	Depletion	bdl	bdl	bdl	bdl	bdl	bdl	bdl	bdl	bdl	bdl
	Repopulation	0.05	0.04		0.03	0.22	0.01	n.d.	0.08	0.03	0.02
	Recovery	0.01	0.04		0.04	0.36	0.03	n.d.	0.11	0.07	0.04
	12 months after repopulation	0.10								0.14	0.08
	24 months after repopulation	0.17				0.40					
BAFF (ng/ml)	Baseline	0.71	1.83	3.72	5.94	3.29	3.05	1.96	3.28	1.33	2.59
	Depletion	0.80	5.50	7.72	7.20	n.d.	5.40	n.d.	6.97	2.45	5.21
	Repopulation	1.79	n.d.		16.3	4.70	4.08	n.d.	4.54	2.00	4.94
	Recovery	3.98	4.47		14.1	3.03	3.30	1.46	2.60	1.41	3.95
	12 months after repopulation	3.51								1.36	2.85
	24 months after repopulation	2.83				2.53					
APRIL (ng/ml)	Baseline	7.86	17.8	6.40	15.1	0.92	17.8	0	182	0.34	10.6
	Depletion	1.56	3.02	3.15	3.53	n.d.	10.8	n.d.	176	0.99	5.75
	Repopulation	8.62	n.d.		1.56	9.20	5.06	n.d.	93.9	1.24	6.65
	Recovery	11.9	2.45		1.24	22.7	4.49	0	65.2	2.21	5.50
	12 months after repopulation	1.62								40.9	6.46
	24 months after repopulation	1.24				23.8					

APRIL = a proliferation-inducing ligand; BAFF = B-cell activation factor of the tumour necrosis factor family; bdl, below detection limit; F, female; n.d., not determined; SLAM, systemic lupus activity measure; SLE, systemic lupus erythematosus.

**Table 2:** Disease activity, B-cell status, BAFF, and APRIL at all time points in patients with RA

	ID	R1	R3	R7	R9	R11	R14	R15	R16	R17
Age (in years)/gender		38/F	73/M	60/F	64/F	60/F	66/F	63/F	60/F	58/M
DAS28	Baseline	6.78	8.14	5.69	5.81	7.69	6.48	7.4	n.d.	5.52
	Depletion	4.42	5.43	n.d.	3.84	n.d.	n.d.	1.97	1.93	n.d.
	6 months post-treatment	3.14	4.41	2.89	2.51	4.51	3.01			
Percentage of CD19^+ ^B cells	Baseline	1	4	4	8	7.6	1.7	6	9.6	7
	Depletion	bdl	bdl	n.d.	bdl	bdl	bdl	bdl	bdl	bdl
	6 months post-treatment	0.5	bdl	bdl	bdl	1				
CD19^+ ^B cells × 10^9 ^per litre	Baseline	0.02	0.05	0.04	0.14	0.19	0.01	0.06	0.18	0.08
	Depletion	bdl	bdl	n.d	bdl	bdl	bdl	bdl	bdl	bdl
	6 months post-treatment	bdl	bdl	bdl	bdl	0.01	bdl			
BAFF (ng/ml)	Baseline	1.01	3.42	0.92	0.75	1.33	1.31	1.90	2.30	1.08
	Depletion	4.27	7.90	n.d.	2.62	n.d.	4.06	3.12	3.51	4.41
	6 months post-treatment	5.88	6.34	4.46	2.83	1.59				
APRIL (ng/ml)	Baseline	409	77.8	189	5.04	96.7	124	179	23.7	39.5
	Depletion	412	54.3	n.d.	2.53	n.d.	112	160	38.2	61.7
	6 months post-treatment	419	32.0	113	1.12	97.1				

APRIL = a proliferation-inducing ligand; BAFF = B-cell activation factor of the tumour necrosis factor family; bdl = below detection limit; DAS28, disease activity score using 28 joint counts; F, female; M, male; n.d., not determined; RA, rheumatoid arthritis.
